# AI-driven mental health decision support linked to clinician resilience and preparedness

**DOI:** 10.3389/fdgth.2026.1755085

**Published:** 2026-04-22

**Authors:** Margareta-Theodora Mircea, Jessica McFadyen, Ross Harper, Max Rollwage, Tobias U. Hauser

**Affiliations:** 1 Limbic Ltd., London, United Kingdom; 2 Max Planck UCL Centre for Computational Psychiatry and Ageing Research, University College London, London, United Kingdom; 3 Department of Psychiatry and Psychotherapy, Medical School and University Hospital, Eberhard Karls University of Tubingen, Tübingen, Germany; 4 German Center for Mental Health (DZPG), Tübingen, Germany

**Keywords:** AI, assessment, burnout, clinician wellbeing, decision support, digital front door, triage

## Abstract

**Objectives:**

Mental health services are facing unprecedented demand, placing significant pressure on clinicians to conduct timely and effective patient assessments. Rising staff turnover and burnout threatens service quality across many countries. This study examined whether providing clinical information, collected via an artificial intelligence (AI)—enabled decision support tool for mental health assessments in the UK's National Health Service (NHS), was associated with differences in clinician wellbeing and patient assessment performance.

**Method:**

In this observational study, we surveyed mental health clinicians (*N* = 131) from nine NHS Mental Health Talking Therapies services on how the information provided by an AI-based decision-support tool related to their experience with conducting clinical assessments. Clinicians reported on assessments where information from the AI tool was available, as well as when it was not (e.g., general practitioner referrals or telephone intakes). Outcomes included clinician wellbeing, task performance, and cognitive load during assessments, with additional analyses assessing the influence of moderating factors, such as clinician experience, workload, and exposure to the tool.

**Results:**

Relative to traditional methods, assessments supported by information provided by the AI tool were associated with significantly higher clinician wellbeing and task performance, and significantly lower cognitive load, irrespective of the clinician's experience. These associations were magnified by workload.

**Conclusion:**

These findings provide preliminary evidence that AI-powered pre-assessment tools may be associated with differences in clinician experience including higher wellbeing, higher task performance, and lower cognitive burden. By targeting systemic drivers of burnout, such tools may represent a potentially scalable approach to support workforce sustainability and service quality in mental health care.

## Introduction

Mental health services are experiencing unprecedented demand, with dramatic increases of up to 40% compared to pre-pandemic levels (1). This is further exacerbated through above-average staff turnover in mental health services and disproportionately high vacancy rates (2). Due to budgetary constraints across many healthcare services worldwide, this directly translates to mounting pressures on mental health services, creating unsustainable workloads for clinicians.

Large caseloads and insufficient organisational support are well-established predictors of reduced wellbeing and heightened emotional exhaustion, the core features of burnout (3, 4). Burnout, in turn, directly compromises patient care: only 28.3% of patients treated by burnt-out therapists demonstrate meaningful improvement, compared with 36.8% of those treated by therapists without burnout (5–7). Taken together, these findings highlight that safeguarding clinician wellbeing is not only essential for workforce sustainability but also for ensuring the effectiveness of psychological treatment.

A case example is the UK's National Health Service (NHS). Within NHS Talking Therapies, Psychological Wellbeing Practitioners (PWPs) are particularly susceptible to burnout, with evidence indicating that elevated work demands substantially contribute to this risk (8, 9). PWP trainees appear especially vulnerable, reporting higher levels of stress and burnout than both their more experienced colleagues and the wider healthcare workforce (10). One contributing factor may be the relatively brief training period before trainees begin clinical practice, which can limit their knowledge and confidence in therapeutic delivery (11). As knowledge and confidence are recognised protective factors, this suggests that enhancing preparedness—during training or through other means—may help reduce burnout risk (3).

Here, we examined whether scalable tools that leverage artificial intelligence (AI) might support clinician preparedness and address factors associated with burnout risk. Virtual triage tools have been proposed as scalable and effective mechanisms to reduce the cognitive and emotional burden on therapists by supporting clinical tasks such as diagnoses, risk assessment, and treatment planning (12). Studies comparing virtual symptom assessment systems with clinicians have shown that some tools can approach human-level performance in diagnostic accuracy and urgency guidance, supporting their feasibility for use in healthcare entry pathways [e.g., (13, 14)]. However, much of this literature has focused on patient-facing outcomes, such as diagnostic accuracy, safety, and service efficiency. Very little is known about how the structured information generated by such tools is experienced by clinicians. Moreover, most existing systems are rule-based and operate within fixed protocols (15, 16), limiting their ability to capture the complexity of patient presentations. A key promise of AI is to expand such capabilities substantially. AI-driven inference engines can adapt dynamically to patient input, medical history, and risk factors, mirroring the flexible reasoning of human clinicians (17).

Here, we investigate an AI decision support tool called Limbic Access, a certified UK CE-marked Class IIa medical device that facilitates chatbot-based patient intake, initial clinician-led assessments, and overall decision support for clinical and administrative staff. This tool interprets symptoms, evaluates risk, and prioritises relevant clinical needs with diagnostic accuracy that surpassed human assessment in past studies (17). Moreover, it has been shown to successfully increase patient access to healthcare (18), streamline clinical workflows (19), increase efficiency of healthcare provision (20), and improve patient recovery rates (19, 20). While prior evaluations of this tool have primarily focused on patient- and service-level outcomes, comparatively little is known about how this tool relates to clinicians' day-to-day experience of conducting assessments, including their wellbeing, perceived performance, and cognitive load. This represents an important gap, as clinician strain and burnout are key constraints on service capacity and quality.

To address this gap, we investigated whether the use of this decision support tool was associated with differences in clinician-reported wellbeing, task performance, and cognitive load during initial patient assessments. As this tool equips clinicians with structured patient information in advance of appointments, we hypothesised that it would be associated with lower cognitive and emotional burden on the clinician performing the assessment (12, 21) and higher self-perceived task performance.

## Materials and methods

### Study design

We employed a within-subjects, comparative survey design to evaluate the impact of the AI decision support tool on clinician wellbeing, task performance and cognitive load. We asked PWPs from nine different NHS Talking Therapies services across England to participate. NHS Talking Therapies services (formerly known as Improving Access to Psychological Therapies, or IAPT) are a UK government initiative delivering evidence-based psychological interventions within a stepped-care model. They primarily serve adults experiencing common mental health difficulties such as anxiety disorders and depression and offer a range of talking therapies treatments (e.g., guided self-help, cognitive-behavioural therapy, counselling, and interpersonal therapy).

In an online survey, clinicians were asked to report their experiences with patient assessments in which they had been provided with clinical information from the AI decision support tool (available only when patients had self-referred through the tool) vs. when this information had not been provided (for patients referring to the service through other means, such as referrals from their GP or self-referring over telephone). This within-subjects comparative approach, where clinicians reported on their experiences with both types of information sources, allowed each PWP to serve as their own control, thereby reducing between-clinician variability and improving sensitivity to differences associated with the information source.

### Participants

A cohort of 146 PWPs were recruited for the study. They were invited to participate by members of the study research team during routine team meetings held within each NHS service. During these meetings, an overview of the study was provided, and interested PWPs were given a direct link to the online survey. As an incentive, participants were entered into a prize draw for a £10 Amazon voucher. All participants provided informed consent online before commencing the survey. This study received ethical approval from NHS London—Surrey Borders Research Ethics Committee (22/PR/1424). The survey was administered as a one-time, cross-sectional online questionnaire. Participants completed the survey asynchronously at a time of their choosing, with no follow-up assessments. Responses were collected between April 2024 and April 2025.

The primary inclusion criterion was being currently employed as a PWP and actively conducting initial patient assessments within one of the participating services. Exclusion criteria were: (a) not having conducted any initial patient assessments in the 30 days preceding survey completion (*n* = 2), or (b) within the preceding 30 days, having *only* conducted assessments informed by the AI decision support tool, or conversely, *only* assessments informed by traditional methods. This latter criterion was essential to ensure participants could provide valid within-subjects comparisons based on recent experience with both types of information sources. Thirteen participants were excluded due to only having done assessments for patients with AI decision support tool information (*n* = 8) or only assessments without this information (*n* = 5), leaving a final sample of 131 participants.

### Materials

#### AI decision support tool

**Figure 1:**
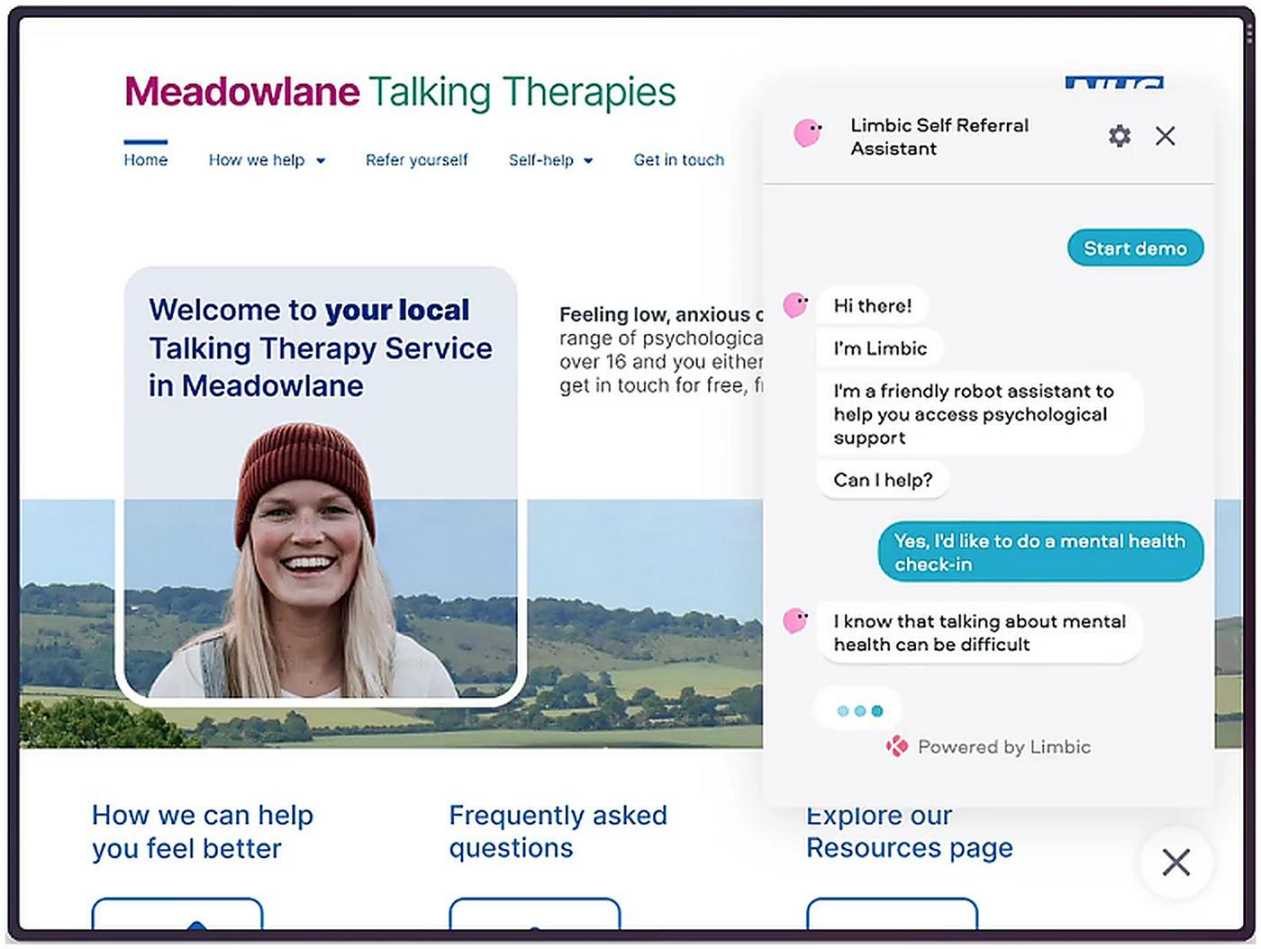
Conceptual mock-up of the AI-powered mental health chatbot as it might appear on a service's landing page during patient self-referral.

The tool evaluated in this study was Limbic Access (https://limbic.ai/access), a commercially available, AI-powered digital product designed to function as an intelligent decision support tool for mental health services (Figure 1). It functions as a chatbot positioned on a mental health provider's web page, where new patients can self-refer easily and discreetly. Patients interact with Limbic Access through a conversational user interface (chatbot) and input information via free text, buttons, and checkboxes. The chatbot uses machine learning to make clinical inference about the most likely diagnoses of a patient and the most relevant associated symptoms, which is used to intelligently guide the referral process and collect valuable clinical patient information that is then summarised and conveyed to the clinical team.

For clinicians, the key feature is the structured clinical referral report that is automatically generated and integrated into the service's electronic health record (EHR). Each report begins by clearly identifying it as a Limbic referral and includes the patient's risk level. Patients that indicate high levels of acute risk (e.g., suicidal intent) are immediately signposted to emergency services and a “crisis alert” is sent to the service. The tool also performs eligibility checks, such as confirming the patient resides within the geographical catchment area of the specific NHS service. The core clinical information then follows in a structured sequence: the AI-inferred primary and secondary presenting problems, the patient's stated expectations from support, “caseness” status (i.e., symptoms above a clinical threshold) across relevant diagnoses, and the condition the patient identified as their primary concern.

Standardised questionnaire—e.g., Patient Health Questionnaire-9 (PHQ-9; 22) for depression and Generalized Anxiety Disorder-7 (GAD-7; 23) for general anxiety—scores are presented to the clinician in an accessible format. A key intelligent feature of the AI decision support tool is the adaptive selection of additional symptom questionnaires for specific anxiety disorders (e.g., social anxiety, panic disorder), which are triggered by the AI during the patient referral process when clarification is needed to resolve uncertainty over the most likely presenting problems. This adaptive, personalised approach tailors the referral process to each patient by presenting only the most relevant questions, thereby improving efficiency and enhancing the accuracy of the inferred presenting problems provided to clinicians.

The report ends with the patient's contact details and demographic information. This structured output provides clinicians with a comprehensive, pre-synthesised overview that extends beyond raw data capture. This clinical information which is automatically collected by the AI decision-support tool, enables the clinicians with a more comprehensive clinical profile of the patient before the clinical assessment which allows for a more in-depth preparation of the assessment. Rather than expending time and effort on information gathering, clinicians can direct their attention to higher-order tasks such as clinical reasoning, therapeutic engagement, and collaborative care planning.

### Traditional referral pathways

Comparator information sources included existing referral methods routinely used within the participating services, including self-referral (e.g., telephone call, static online forms on the service's website, walk-ins), referrals from primary care (e.g., general practitioners), referrals from secondary care (e.g., specialist mental health services), and referrals from other agencies (e.g., social services). All participating services used EHR systems as part of routine clinical practice. However, for patients entering through traditional referral routes, the information recorded in the EHR at the point of assessment typically comprised basic administrative details and brief, often unstructured free-text descriptions, with limited standardised symptom or risk data. The AI decision support tool operates upstream of the EHR, collecting structured, patient-reported information via an adaptive conversational interface and generating a synthesised clinical summary that is then integrated into the EHR before the assessment. It also provides clinicians with the most likely primary and secondary presenting problems, determined using its proprietary machine learning algorithms. The incremental value therefore lies not simply in digitisation, but in the standardisation, completeness, and automated clinical inference applied to intake information.

### Survey instrument

A custom online survey was developed to capture participants' comparative experience with patient assessments informed by the AI decision support tool vs. those informed by information from traditional referral pathways. The survey first collected information about the participant's professional role (e.g., job title, employment duration) and frequencies of patient assessments per week in the last 30 days. Participants then indicated the proportion of recent patient assessments for which they viewed information derived from the AI decision support tool (5-point Likert scale from “none” to “all”).

The core of the survey employed a structured comparative approach to assess key outcomes. These were administered in two distinct stages: first, an item set regarding assessments *with* the information provided by the AI decision support tool, covering clinician wellbeing, task performance and cognitive load (see **Outcome Measures**); second, the identical item set but instead based on experiences conducting assessments *without* the AI decision support tool-derived information (i.e., patient intakes through traditional referral methods).

All survey items required clinicians to reflect on their own practice over the preceding 30 days. As surveys were completed asynchronously, the specific 30-day reference period differed across participants. The study therefore captures clinicians' recent experiences relative to their individual survey completion dates, rather than a fixed calendar window.

### Outcome measures

The primary outcome measures were clinician wellbeing, task performance, and cognitive load, all assessed comparatively for experiences with assessments informed by the AI decision support tool vs. those with information from traditional referral pathways. All questions used to assess the outcome measures are provided in the Supplementary Material.

### Clinician wellbeing

Six items assessed clinicians' affective states related to conducting assessments. These included both positive affect (feelings of energy, confidence, and comfort) and negative affect (stress, uncertainty, and anxiety). Each question was phrased as, “How [emotion] do you feel about these assessments?” where the emotion is the outcome measure (e.g., “stressed”) and “these assessments” refers to either those with AI decision support tool information provided or those without. Responses to all items were made on a 7-point Likert scale from “not at all” to “extremely”. Negative affect items were reverse scored, and all items were averaged to produce a single wellbeing measure.

### Task performance

Seven items evaluated the perceived ease of performing key assessment-related tasks. These included preparing for the assessment, determining appropriate treatment, conducting a risk assessment, identifying a diagnosis, completing the assessment within the appointment time limit, building a patient relationship, and managing patient expectations. Each question was phrased as, “How easy is it to [task]?” where “task” is the variable (e.g., “complete an assessment”). Responses were made on a 7-point Likert scale from “not at all” to “extremely”, with responses averaged across items to produce a single task performance score.

Internal consistency was assessed using Cronbach's *α*, a standard measure of reliability for multi-item Likert scales that evaluates the degree to which items measure a common underlying construct (24). Reliability was good for both bespoke scales: wellbeing (α = 0.844) and task performance (α = 0.884).

### Cognitive load

Cognitive load was measured using a modified version of the NASA Task Load Index (NASA-TLX; 25). The NASA-TLX has demonstrated strong internal consistency (e.g., Cronbach's α = 0.80; 26) and moderate convergent validity with other workload measures (r = 0.33 to 0.49; 26, 27).

Adaptations for this study involved removing the “physical demand” subscale, as it was not relevant to the task, and omitting the pairwise weighting procedure for item pairs to reduce complexity. The modified scale comprised five items assessing: mental demand (“How mentally demanding is the assessment?”), hurried pace (“How hurried or rushed is the pace of the assessment?”), success in accomplishment (“How successful are you in accomplishing what you want to accomplish?”), effort (“How hard do you have to work to accomplish your level of performance?”), and negative affective response (e.g., insecurity, irritation, stress; “How insecure, discouraged, irritated, stressed, or annoyed do you feel?”). Responses were made on a 7-point Likert scale from “not at all” to “extremely” (adapted from the original 11-point scale to maintain consistency across all outcome measures administered in the survey), and the item “success in accomplishment” was reverse-scored. Responses were averaged across items to produce a single cognitive load score.

### Statistical analysis

Linear mixed-effects regression models were used to examine the impact of the AI decision support tool on clinician wellbeing, task performance and cognitive load, with each outcome predicted by a separate model. All analyses were conducted in Python (version 3.11) using the statsmodels package (version 0.14.3). Each clinician contributed data under two conditions (AI decision support tool-based assessments vs. traditional methods of referral-based assessments), resulting in 262 observations per model. A random intercept was included for each clinician. All continuous outcomes and predictors were standardised (z-scored) to allow effect size inferences from the resultant coefficients (β). To further explore item-level effects within each averaged outcome variable, paired *t*-tests were conducted for each specific questionnaire item, with *P*-values corrected for multiple comparisons using the false discovery rate (FDR) method.

Formally, for clinician *i* under referral condition *j*, models took the form:

$$\text{z}\_ij=\beta_{0}+\beta_{1}\;\text{Referral}\_\text{ij}+\beta_{2}\;\text{Experience}\_\text{i}+\beta_{3}\;\text{Workload}\_\text{i}+\beta_{4}\;\text{Exposure}\_\text{i}+\gamma \_\text{sSite}\_\text{s}+\text{u}\_\text{i}+\epsilon \_\text{ij}$$

where *z_ij* denotes the standardised outcome score (wellbeing, task performance, or cognitive load). *Referral_ij* is a binary indicator denoting assessments supported by the AI decision support tool vs. traditional referral information. *Experience_i* represents ordinal employment duration, *Workload_i* captures the number of patient assessments conducted per week, and *Exposure_i* reflects the proportion of recent assessments supported by the AI tool. Fixed effects for service site (*Site_s*) were included to account for between-site differences. A clinician-specific random intercept (*u_i*) was included to account for repeated observations within clinicians, and *ε_ij* represents the residual error term. Interaction terms between referral condition and each covariate were added in separate models, as described above. As a robustness check, we additionally estimated clinician fixed-effects models using within-clinician first differences, which remove all time-invariant clinician characteristics.

In all models, we controlled for three potentially moderating variables: *experience* (employment duration), *workload* (number of patient assessments per week) and *exposure* (proportion of assessments in which clinicians received referral information from the AI decision support tool). Employment duration was included as an ordinal variable to determine whether the tool was equally effective for early-career and more experienced clinicians. The average number of patient assessments per week was used as a continuous variable, indicating workload intensity. Finally, the reported proportion of assessments in which clinicians received AI decision support tool referral information was included as an ordinal variable (“some”, “half”, or “most” assessments per week) to assess whether the frequency of exposure influenced perceived benefits.

For each of the three outcome variables, four models were estimated, yielding a total of twelve models. The first model included the main effects of the AI decision support tool and all three covariates (experience, workload, and exposure). The remaining three models each included an interaction term between the AI tool variable and one of the covariates, while controlling for the remaining covariates. This approach allowed us to test whether the impact of the AI decision support tool varied as a function of each covariate, while minimising multicollinearity among predictors. Model fit was assessed using Akaike Information Criterion (AIC) and Bayesian Information Criterion (BIC), with lower values indicating better relative fit.

## Results

We conducted a within-subjects comparative survey with 131 PWPs from nine NHS Talking Therapies services to assess how an AI decision support tool was associated with differences in clinician wellbeing, task performance, and cognitive load, while accounting for clinician experience, workload, and exposure to the tool.

### Clinician characteristics

All 131 participants were PWPs (91.6% full-time) with a range of experience levels and seniority. Professional tenure was skewed toward early-career practitioners, with 90.8% being in post for under five years, including 1.5% for 1–3 months, 7.6% for 3–6 months, 15.3% for 6–12 months, 32.1% for 1–2 years, and 34.4% for 2–5 years. A smaller group had more than five years of experience (6.1% for 5–10 years, 3.0% for over 10 years). Clinicians reported conducting an average of 8.47 client assessments per week (SD = 5.3, range = 1 to 36).

Over half of clinicians (58.8%) reported that most of their assessments were supported by the AI decision support tool, and nearly a quarter (26.7%) reported roughly an equal split between referrals with and without AI tool information. Only 14.5% of clinicians reported that just some of their assessments were guided by the AI tool output. These varied usage figures provide a solid basis for evaluating the impact of the AI decision support tool in routine practice.

In line with the service evaluation focus of the study and to minimise identifiable information within relatively small clinical teams, we did not collect clinicians' personal demographic variables such as age or gender. Consequently, the sample is described primarily in terms of professional role, employment status, and clinical experience. While this approach supported anonymity and participation, it limits the ability to characterise the sample demographically or explore whether outcomes vary by these factors.

### Higher clinician wellbeing with AI-supported assessments

**Figure 2:**
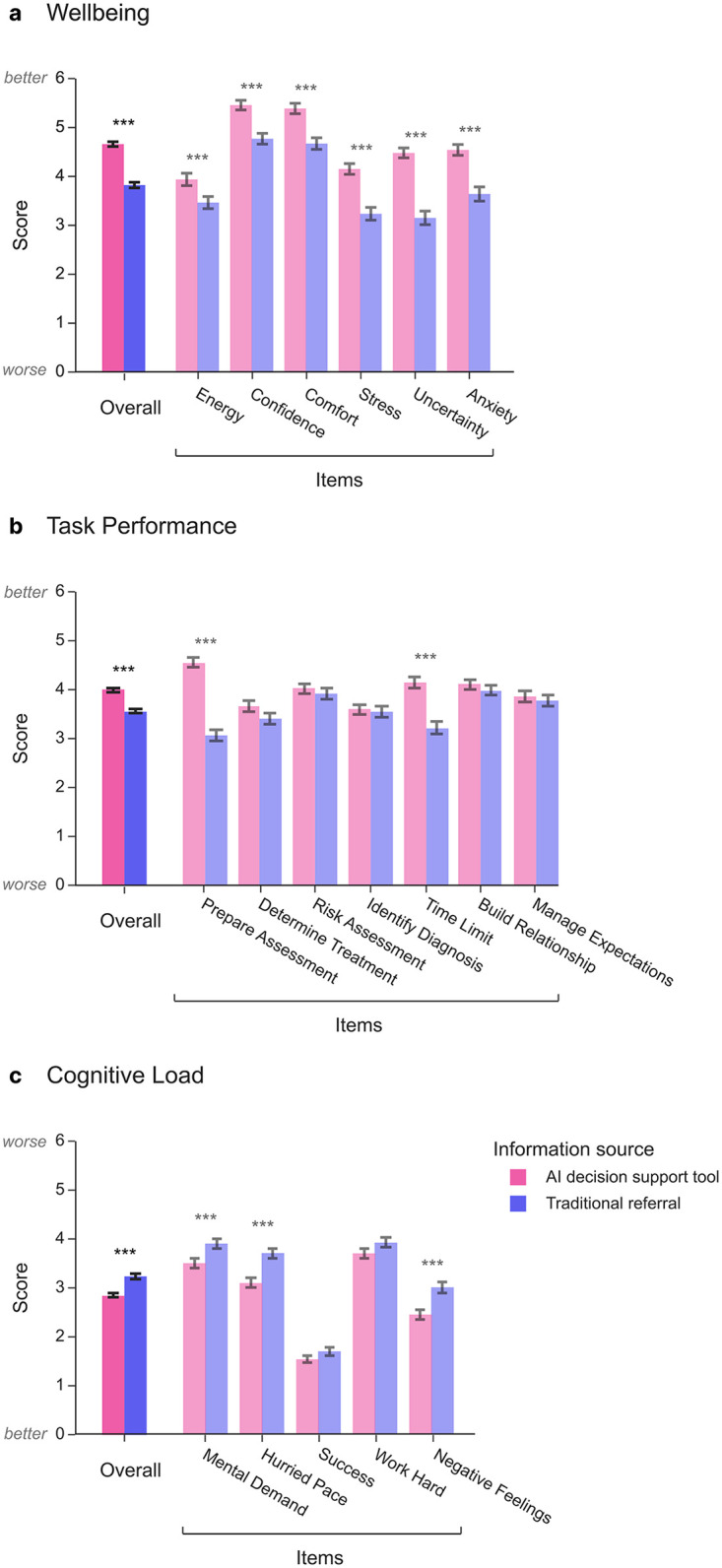
Clinician wellbeing, task performance, and cognitive load outcomes by information source. **(a)** Average wellbeing scores reported by clinicians for patient assessments that had information provided by the artificial intelligence (AI) decision support tool (pink) or for those that had information from traditional referral methods (e.g., general practitioner referrals, telephone intakes; purple). The overall score represents the average of each individual item. Error bars indicate standard error of the mean. Significance indicators for “overall” represents the regression coefficient from a linear mixed effects model for the information source predictor. Significance indicators for each individual item represent the false discovery rate corrected results from a series of paired *t*-tests. * *P* < .05, ** *P* < .01, *** *P* < .001. **(b)** Same as **(a)** except for task performance relating to the patient assessment. **(c)** Same as **(a)** except for cognitive load [adapted NASA Task Load Index (NASA-TLX)], with lower scores indicating reduced (and thereby improved) cognitive load. *N* = 131 clinicians.

Clinician wellbeing is closely tied to burnout risk and service quality (5, 28), and so we first examined whether clinicians reported different levels of wellbeing when assessments were supported by the AI tool. Clinicians were asked to report their energy levels, confidence, comfort, stress, uncertainty and anxiety associated with clinical assessments. Clinicians reported significantly higher wellbeing overall (Figure 2a) when using the AI decision support tool (M = 4.7, SD = 0.9, on a scale from 0 to 6) compared with traditional referral information (M = 3.8, SD = 1.1; β = 0.77, 95% CI = [0.57, 0.97], *P* = 2.32 × 10^−14^). This reflects a large effect, with wellbeing scores approximately 15% higher for assessments supported by the AI tool. This pattern of higher wellbeing scores was seen across all items: increased energy, confidence, and comfort, alongside decreased stressed, uncertainty, and anxiety (all pairwise *t*-tests P_FDR_ < .002).

Out of the control variables, only “exposure” was a significant moderator, where clinicians who performed relatively more assessments using the AI decision support tool also reported larger differences in wellbeing for these assessments relative to those for patients coming through traditional referral methods (β = 0.34, 95% CI = [0.15, 0.52], *P* = 5.15 × 10^−4^). This indicates that greater exposure to AI-generated referral information was associated with higher wellbeing.

### Higher task performance during assessments

As effective assessment underpins accurate diagnosis and treatment planning, we next examined whether clinicians reported differences in task performance during assessments supported by the AI tool. Clinicians rated the ease with which they prepared for assessments, determined treatment pathways, assessed risk, identified diagnoses, adhered to time limits, built relationships with the patient and managed their expectations. Results showed significantly higher task performance scores (Figure 2b) when reporting on assessments supported by the AI tool (M = 4.6, SD = 1.0, on a scale from 0 to 6) compared to traditional referral information (M = 4.0, SD = 1.2), β = 0.42, 95% CI = [0.19, 0.66], *p* = 3.427 × 10^−4^), with a moderate effect size. Follow-up paired *t*-tests revealed this was driven predominantly by feeling more prepared for the assessment (P_FDR_ = 4.10 × 10^−13^) and being more able to complete the assessment within the appointment time (P_FDR_ = 7.67 × 10^−9^).

These higher task performance ratings during AI-supported assessments were accentuated for clinicians with a higher assessment workload [β = 0.26, 95% CI = (0.03, 0.49), *P* = 0.026] and for those exposed more to the AI reports [β = 0.36, 95% CI = (0.13, 0.59), *P* = 0.002]. Employment duration of the clinicians did not significantly modulate the AI effects [β = −0.23, 95% CI = (−0.46, 0.01), *P* = 0.063], suggesting similar benefits of the AI decision support tool across clinician experience levels.

### Lower cognitive load during AI-supported assessments

Lastly, we assessed whether cognitive load differed between AI-supported and traditional assessments, given the potential for cognitive load to compromise decision-making and increase stress. We measured cognitive load using a modified version of the NASA Task Load Index (NASA-TLX; 25) comprising five items assessing: mental demand, hurried pace, success in accomplishment, effort, and negative affective response.

Clinicians reported significantly lower cognitive load for assessments supported by the AI decision support tool (M = 3.5, SD = 0.9, on a scale from 0 to 6) compared with traditional referral information (M = 3.9, SD = 1.0), β = −0.48, 95% CI = [−0.65, −0.31], *P* = 2.955 × 10^−8^), with a moderate-to-large effect size (Figure 2c). Follow-up paired *t*-tests on each item revealed that the largest differences were observed for the AI tool at reducing mental demand, hurried pace, and negative feelings about the assessment (all P_FDR_ < 3.93 × 10^−4^), while there were no significant difference in the feelings of success or accomplishment, nor working hard, after family-wise error-correction (all P_FDR_ > .182). These findings suggest that cognitive demands ratings were lower during AI-supported assessments across multiple dimensions. These differences in cognitive load were seen more for clinicians who conducted relatively more assessments where an AI-generated report was available [β = −0.18, 95% CI = (−0.35, −0.02), *P* = 0.032].

For clinician wellbeing and cognitive load, the baseline mixed-effects models including referral condition alone yielded the lowest AIC and BIC values (wellbeing: AIC = 705.02, BIC = 715.72; cognitive load: AIC = 704.11, BIC = 714.82), indicating that additional covariates or interaction terms did not improve model fit. For task performance, models including a workload × referral interaction showed a modest improvement in AIC relative to the main-effects specification (AIC = 755.51 vs. 755.98), although BIC continued to favour more parsimonious models due to increased complexity. Taken together, these indices suggest that the primary association between referral condition and clinician outcomes is robust and does not generally depend on additional moderators.

Results from clinician fixed-effects models implemented via within-clinician first differences closely mirrored the mixed-effects findings across all outcomes. Relative to traditional referral information, assessments supported by the AI tool were associated with higher clinician wellbeing [mean difference = 0.84, 95% CI (0.62, 1.05), *P* < .001] and higher perceived task performance [mean difference = 0.50, 95% CI (0.23, 0.76), *P* < .001), as well as lower cognitive load [mean difference = −0.48, 95% CI (−0.64, −0.31), *P* < .001). These results indicate that the observed associations are robust to controlling for all time-invariant clinician characteristics.

## Discussion

This study examined the associations between the use of an AI-powered clinical decision support tool for mental health assessments and clinicians' experience in everyday practice. We found that assessments supported by information from the AI decision support tool were associated with higher clinician wellbeing and task performance and lower cognitive load. Importantly, these associations were evident across varying levels of clinical experience and were particularly pronounced among clinicians with heavier assessment workloads. Notably, the comparison in this study is not between AI and the absence of electronic records, as all services used routine EHR systems, but between standard referral information typically available within those records and the additional structured, synthesised information generated by the AI-supported intake process. In the context of resource-constrained healthcare systems and increasing clinician strain, our results indicate that AI-enabled decision support tools may be associated with improvements in clinician experience in domains closely linked to burnout and service quality.

Clinicians reported significantly better wellbeing when conducting patient assessments with information provided by the AI decision support tool compared to when such information was absent. Typically, clinicians begin assessments with very limited knowledge about a patient's presenting problems, severity, or risk, which can heighten stress during initial consultations. In contrast, the AI-enabled decision support tool offers access to this information in advance, which may reduce uncertainty and may be linked to higher clinician wellbeing. Given that clinician wellbeing is a strong predictor of burnout risk (28–30), these findings suggest that AI-powered decision support tools may support in lowering burnout risk and its downstream effects on service quality and patient outcomes (5–7).

Self-reported performance during assessments was also significantly higher when clinicians used the AI decision support tool, with the most notable differences observed in their ability to prepare for assessments and complete them within the allotted appointment time. This may be because the AI tool provides substantial clinical information—much of which would otherwise need to be gathered during the assessment—as well as decision support that facilitates key clinical inferences such as diagnosis and treatment planning. By supporting decision-making, the tool may free up time for interpersonal engagement between clinician and patient, particularly for rapport-building. Strengthening rapport, a core component of initial assessments, can be especially valuable in high-throughput settings such as NHS Talking Therapies, where limited appointment durations often constrain meaningful connection (31). Because the therapeutic relationship is a robust predictor of recovery and treatment adherence (32–34), this enhancement could have important downstream implications for patient care. Additionally, clinicians reported significantly lower cognitive load when supported by the AI tool. Lower ratings of negative affect, mental demand, and effort, recognised dimensions of cognitive load associated with clinician burnout risk (9), were observed when assessments were supported by the tool.

These findings complement prior studies (19), showing that the benefits of AI-enabled decision support may extend beyond the patient experience (e.g., with accessing mental health services) to also include clinician-reported outcomes. Specifically, prior work has demonstrated reduced therapy drop-out and faster recovery (17, 20), while the present study provides new evidence of higher clinician wellbeing and task performance, and lower cognitive load. This dual impact on both patients and clinicians highlights that AI-powered front door technologies may represent promising tools for supporting patient pathways while safeguarding the workforce, thus addressing the intertwined challenges of patient recovery and clinician burnout in mental health services.

Our findings are of particular interest when considering that individual-level strategies (e.g., mindfulness, stress management) provide only modest and short-lived relief from clinician burnout, whereas systemic organisational interventions—particularly those addressing workload and workflow—are essential for sustained wellbeing (35–37). AI decision support tools may represent one such systemic approach. By supporting clinician wellbeing during initial assessments and potentially reducing task demands, they may influence the underlying drivers of burnout. In doing so, they move beyond the limitations of reactive wellbeing programmes, and contribute to efforts aimed at building a more resilient and sustainable mental health workforce.

Several limitations should be acknowledged. First, wellbeing and task performance were assessed using bespoke scales rather than validated instruments. This choice was made to ensure brevity and alignment with the specific context of NHS Talking Therapies, though it limits comparability with prior research and warrants cautious interpretation given greater measurement uncertainty than established validated instruments. Although both scales showed good internal consistency, replication using established or formally validated instruments would strengthen confidence in the findings. Second, the reliance on self-report may have introduced measurement error and subjective bias. Responses may have been influenced by social desirability and retrospective comparisons across referral types may be affected by recall bias. Prospective or experimental designs would allow stronger causal inferences. In addition, clinicians' perceptions may reflect novelty or habituation effects, whereby initial enthusiasm or increasing familiarity with the tool alters reported experiences over time. The study also lacked objective performance indicators, such as assessment accuracy, duration, or patient outcomes, limiting the ability to corroborate perceived benefits with behavioural or clinical metrics. Future research should incorporate prospective designs and objective measures (e.g., experience sampling, time tracking, or transcript-based performance analysis) to strengthen causal inference and triangulate findings.

Generalisability may be limited by the sample composition. Participants were exclusively PWPs within NHS Talking Therapies services and were predominantly early-career, with over 90% reporting fewer than five years' experience. Although this distribution reflects the typical workforce profile of many Talking Therapies services, findings may therefore not directly extend to more experienced clinicians or other professional groups, such as psychologists or psychiatrists, whose roles and decision-making processes may differ. More senior clinicians may rely less on structured pre-assessment information due to greater clinical expertise, or conversely may derive different efficiency benefits. In addition, personal demographic data (e.g., age, gender) were not collected to minimise respondent burden and protect anonymity within small teams, restricting demographic characterisation and subgroup analyses. Future research should examine more experienced, multidisciplinary, and demographically described samples.

A further limitation relates to the observational assignment of patients to referral pathways (AI-supported vs. traditional). Because patients self-selected or were directed into different referral routes, allocation was not randomised and we did not have access to patient-level demographic or clinical data to assess comparability across pathways. As a result, systematic differences in case-mix may have been present. For example, patients using the AI pathway may differ in age, digital literacy, symptom severity, or complexity of presentation relative to those referred through traditional routes. Such differences could influence clinicians' perceived workload and experiences independently of the tool itself, introducing potential confounding. Consequently, observed effects may reflect not only the analytical benefits of the AI system but also differences in the completeness or structure of information collected at intake. Although prior evaluations of the same tool have not identified clear population differences between pathways (19), residual selection bias cannot be ruled out. Future research should link clinician- and patient-level data or employ randomised or quasi-experimental designs to more rigorously isolate causal effects.

## Conclusion

Our findings suggest that AI-powered decision support may represent a promising method for helping address acute workforce pressures experienced by mental health practitioners in the UK, where nearly one-third of healthcare staff report time off for mental health reasons (4), a striking indicator of systemic strain. Mental health services experience consistently higher turnover than sector-wide averages, as well as higher vacancy rates (2), indicating that recruitment alone is not a sustainable solution. High task demands are strongly associated with impaired clinician wellbeing (3, 8, 9) and reduced patient recovery rates (5–7). Similar challenges are observed internationally, with burnout rates for US clinicians approaching 50% (38), turnover in mental health roles as high as 22%–35% annually (39), and absences from work due mental health issues increasing by 300% from 2017 to 2023 (40). Our findings therefore suggest that some of these pressures could be, at least in part, addressed by supporting clinicians with intelligent digital support tools.

Such workforce pressures directly undermine service quality and continuity, creating a vicious cycle of burnout and inefficiency (21). Against this backdrop, scalable systemic interventions are urgently needed. Intelligent AI-powered tools, like the one studied here, may offer a potential solution to cognitive and emotional strain and workflow inefficiencies. In doing so, such tools may support efforts to address key drivers of staff turnover, contribute to a more resilient and sustainable mental health workforce, and potentially support patient pathways to recovery.

## Data Availability

The datasets presented in this study can be found in online repositories. The names of the repository/repositories and accession number(s) can be found below: https://github.com/LimbicAI/study-2025-clinician-preparedness.
